# High insecticide resistance in the major malaria vector *Anopheles coluzzii* in Chad Republic

**DOI:** 10.1186/s40249-019-0605-x

**Published:** 2019-12-03

**Authors:** Sulaiman S. Ibrahim, Amen N. Fadel, Magellan Tchouakui, Ebai Terence, Murielle J. Wondji, Micareme Tchoupo, Clement Kérah-Hinzoumbé, Samuel Wanji, Charles S. Wondji

**Affiliations:** 1 Vector Biology Department, Liverpool School of Tropical Medicine (LSTM), Liverpool, L3 5 QA UK; 2 Department of Biochemistry, Bayero University, Kano, PMB 3011 Nigeria; 3 Centre for Research in Infectious Diseases (CRID), LSTM Research Unit, P.O. Box 13591, Yaoundé, Cameroon; 4 Department of Microbiology and Parasitology, University of Buea, P.O. Box 63, Buea, Cameroon; 5 National Malaria Control Program, P.O. Box 2030, N’djamena, Chad

**Keywords:** *Anopheles coluzzii*, Malaria, Sahel, Chad, Insecticide, Resistance, Metabolic, *Kdr*

## Abstract

**Background:**

The Sahel region of Chad Republic is a prime candidate for malaria pre-elimination. To facilitate pre-elimination efforts in this region, two populations of *Anopheles coluzzii* from Central Chad Republic were characterized, their insecticide resistance profile and the possible molecular mechanisms driving the resistance in the field investigated.

**Methods:**

Bloodfed female *Anopheles gambiae* s.l. resting indoor, were collected at N’djamena and Massakory, Chad in 2018 and characterized for species composition, and infection rate was determined using the TaqMan assay. Susceptibility to various insecticides was assessed using WHO tube bioassays. Cone bioassays were conducted using various long-lasting insecticidal nets (LLINs). Results were analysed using Chi Square test. Knockdown resistance (*kdr*) and *ace-1* markers were investigated by TaqMan genotyping.

**Results:**

*Anopheles coluzzii* was the major vector found in N’djamena (100%) and Massakory (~ 94%). No *Plasmodium* was found in 147 bloodfed F_0_ *An. coluzzii* (82 from N’djamena and 65 from Massakory)*.* High intensity pyrethroid resistance was observed with mortalities of < 2% for permethrin, deltamethrin and etofenprox, and with < 50% and < 60% dead following exposure to 10× diagnostic doses of deltamethrin and permethrin, respectively. For both sites, < 10% mortalities were observed with DDT. Synergist bioassays with piperonylbutoxide significantly recovered pyrethroid susceptibility in Massakory populations, implicating CYP450s (mortality = 13.6% for permethrin, *χ*^2^ = 22.8, df = 1, *P* = 0.0006; mortality = 13.0% for deltamethrin, *χ*^2^ = 8.8, df = 1, *P* < 0.00031). Cone-bioassays established complete loss of efficacy of the pyrethroid-based LLINs; and a 100% recovery of susceptibility following exposure to the roof of PermaNet®3.0, containing piperonylbutoxide. Both populations were susceptible to malathion, but high bendiocarb resistance was observed in Massakory population. The absence of *ace-1* mutation points to the role of metabolic resistance in the bendiocarb resistance. Both 1014F and 1014S mutations were found in both populations at around 60% and < 20% respectively. Sequencing of intron-1 of the voltage-gated sodium channel revealed a low genetic diversity suggesting reduced polymorphism.

**Conclusions:**

Multiple resistance in *An. coluzzii* populations from Chad highlight challenges associated with deployment of LLINs and indoor residual spraying (IRS) in the Sahel of this country. The pyrethroid-synergists LLINs (e.g. PermaNet®3.0) and organophosphate-based IRS maybe the alternatives for malaria control in this region.

## Multilingual abstracts

Please see Additional file 1 for translations of the abstract into the five official working languages of the United Nations.

## Background

Malaria causes significant morbidity and mortality each year [1]; with the WHO African Region accounting for ~ 92% of all malaria-related deaths [2]. For almost two decades the global cases of malaria have been declining, with few endemic countries even transitioning towards elimination [3]. However, this decline in malaria cases stalled between 2015 and 2017 [2, 4] with cases even increasing globally [4, 5]. This rebound in malaria transmission is a warning sign that primary regions of interests for sustained control and pre-elimination need urgent attention [6]. Chad is one of such areas, characterised with a high seasonal malaria transmission [7]; and ~ 80% of its population living in high malaria transmission areas. Of the 14.9 million people living in Chad less than 50% have access to the core malaria control tools-the long-lasting insecticidal nets or indoor residual sprays [2]. It is not surprising that Chad is among the 18 countries which account for ~ 80% of global malaria deaths [2]. The Sahelian region of Chad, representing the northern limit of malaria endemicity in the country, is important for pre-elimination due to seasonality in transmission of malaria. However, more information on molecular basis of insecticide resistance from vectors in this region is required to facilitate evidence-based planning and implementation of control measures.

The major malaria parasite in Chad is the deadly *Plasmodium falciparum,* which account for ~ 100% of all parasite detected [2]; and the major malaria vectors have been reported as *Anopheles arabiensis* and *An. gambiae* s.s. (M and S forms) [8–10]. Several studies have established insecticide resistance in the *An. arabiensis* and *An. gambiae* s.s. from Chad and described the molecular basis of the resistance. For example, increased pyrethroid resistance in *An. gambiae* s.l. populations from south-west Chad [9]; pyrethroid, dichlorodiphenyltrichloroethane (DDT) and bendiocarb resistance in *An. gambiae* s.l. populations from Kome, southern Chad [8]. Presence of 1014F knockdown resistance (*kdr*) mutation has also been established in the various *An. gambiae* s.s. populations from southern Chad, and its limited presence in the *An. arabiensis* populations which predominate in the north [8, 11]. However, little is known of the malaria vectors in the drier Sahel regions of the country north of N’djamena. For proper implementation of vector control in the Sahel of Chad, more information is required on composition of the major malaria vectors from the region, their role in transmission, their insecticide resistance profiles and the various mechanisms driving the resistance in the field.

Here, we report a primary data on two populations of the major malaria vector *An. gambiae* s.l. from Sahel region of Chad. The role of these vectors in malaria transmission, their resistance status to various public health insecticides, and the underlying molecular mechanisms driving the resistance in the field was investigated.

## Methods

### Study site and sampling

**Fig. 1:**
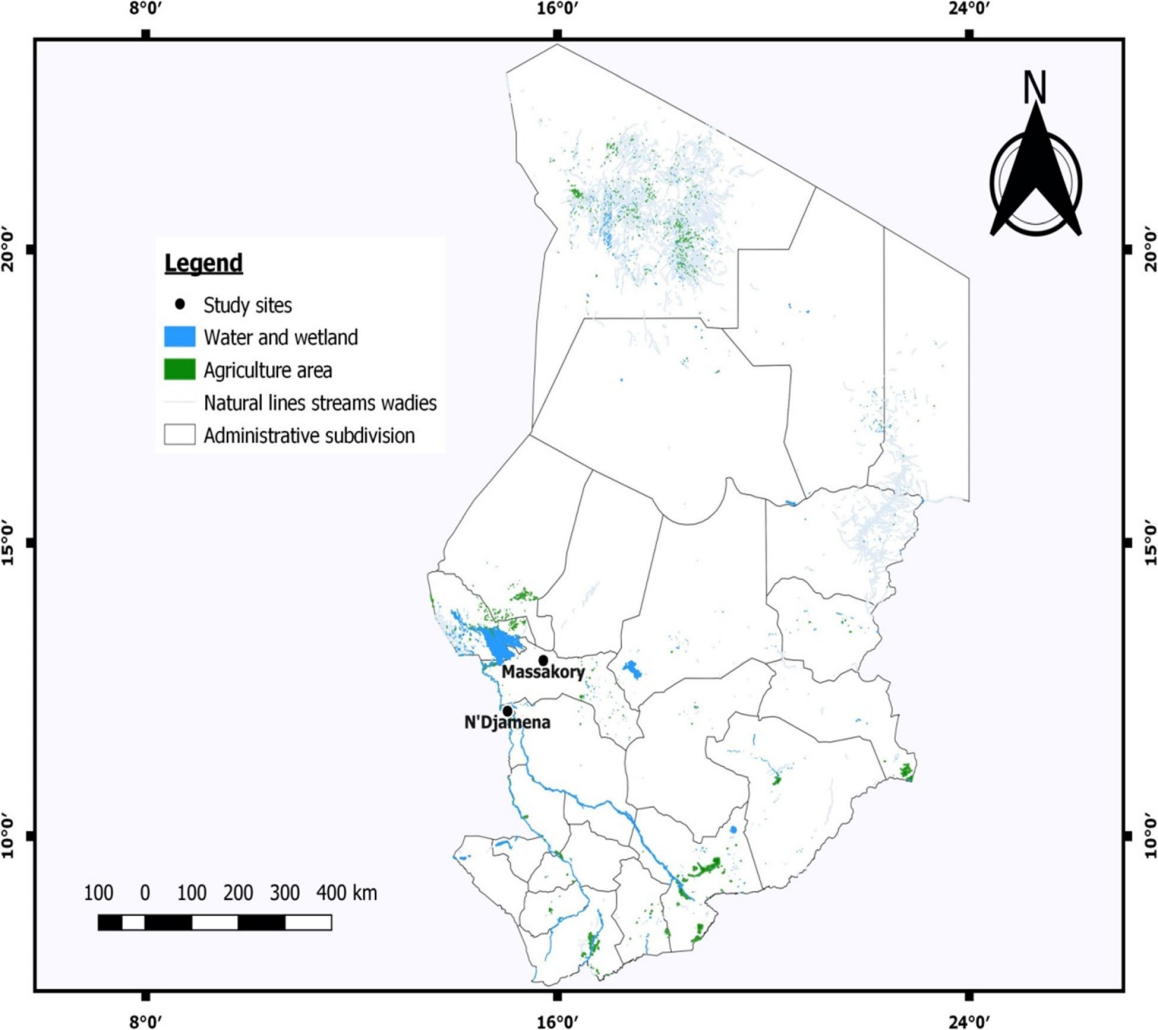
A map showing the two sampling sites in the Sahel of Chad

The Ministry of Public Health of Chad, through the National Malaria Control Program (NMCP) provided authorization for field work at N’djamena and Massakory (Clearance Number: 423/PR/MSP/DG/PNLP/2018). Mosquitoes were collected indoor, in the early hours of morning (6:00 am–8:00 am), using battery-powered aspirators (John. W. Hock, Florida, USA). Collection was conducted from randomly selected houses in N’djamena (12° 6′ N, 15° 02′ E) and Massakory (12° 59′ N, 15° 43′ E) between 14th to 22nd of August 2018 (Fig. 1). With annual rainfall of ~ 400 mm, the Logone River, within Chari drainage basin of N’djamena allows year-round cultivation of vegetables with associated application of insecticides, notably pyrethroids, carbamates and organophosphates, to protect crops (http://www.reca-niger.org/IMG/pdf/-4.pdf). Massakory, with no permanent water body is in the arid Sahelian belt, north-east of N’djamena and is the capital of Chadian region of Hadjer-Lamis. Bloodfed female mosquitoes were maintained on 10% sugar at 25 ± 2 °C and 70–75% relative humidity until fully gravid. They were transferred individually into 1.5 ml tubes and forced to lay eggs [12]. All F_0_ parents identified as belonging to *An. gambiae* complex using morphological keys [13] and confirmed as *An. coluzzii* using the SINE200-PCR [14] were allowed to lay eggs. Egg batches were transferred into paper cups for hatching in the Centre for Research in Infectious Diseases (CRID), Yaounde’, Cameroon. Eggs were pooled into bowls and supplemented with Tetramin™ baby fish food. All F_1_ females that emerged were randomly mixed in cages and 2 to 4-days old were used for insecticide bioassays.

### *Anopheles* species identification

Following morphological identification, genomic DNA was extracted from the female *Anopheles* which laid eggs, using the Livak protocol [15]. Species identification to the molecular level was conducted using the SINE200 PCR [14].

### Estimation of sporozoite infection rate

To establish infection with *Plasmodium,* 147 *An. gambiae* s.l. females (82 from N’djamena and 65 from Massakory) that laid eggs were dissected, head/thoraces used for DNA extraction and TaqMan assay [16], with MX 3005 (Agilent, Santa Clara, USA). 1 μl of gDNA was used for amplification with the following condition: initial denaturation at 95 °C for 10 min, followed by 40 cycles each of 15 s at 95 °C and 1 min at 60 °C. Primers described previously [PlasF_GCTTAGTTACGATTAATAGGAGTAGCTTG and PlasR_GAAAATCTAAGAATTTCACCTCTGACA [16]] were used together with two probes labelled with fluorophores, FAM (Falcip+_TCTGAATACGAATGTC) to detect *Plasmodium falciparum*, and HEX (OVM + _CTGAATACAAATGCC) to detect combination of *P. ovale, P. vivax* and *P. malariae*. Positive samples (known FAM+ and OVM+) were used as controls, in addition to a negative control to which 1 μl of ddH_2_0 was added. TaqMan assay results were validated using a nested PCR [17]. Sporozoite rate was calculated as percentage of mosquitoes with sporozoites in comparison to the total number of the females examined [18].

### WHO insecticide susceptibility tests

Bioassays were performed following the WHO protocol [19] with representative insecticides from the four major public health classes. For N’djamena, seven insecticides were tested: (i) the type I pyrethroid: permethrin (0.75%); (ii) the type II pyrethroid: deltamethrin (0.05%); (iii) a pseudo-pyrethroid: etofenprox (0.5%); (iv) the organochloride: DDT (4%); (v) the carbamates: bendiocarb (0.1%) and propoxur (0.1%); and (vi) one organophosphate: malathion (5%). For Massakory, permethrin, deltamethrin, DDT, bendiocarb and malathion were tested. Insecticide impregnated papers (reference: WHO/VBC/81.806) were sourced from the WHO/Vector Control Research Unit (VCRU) of University of Sains Malaysia (Penang, Malaysia). Four replicates of 20–25 F_1_ females (2–4 days old) per tube were used for each insecticide. Mosquitoes were transferred from holding tubes to tubes lined with insecticide papers and exposed for 1 h. After 1 h exposure, mosquitoes were transferred back to the holding tubes, supplied with 10% sugar and mortality recorded at 24 h. For each bioassay one replicate of 20–25 females unexposed to any insecticides were used as control. To confirm the integrity of insecticide papers, the fully susceptible *An. coluzzii* (Ngoussou colony) [20] was tested alongside the field populations. Mosquitoes were considered susceptible to an insecticide where mortality was > 98%, suspected to be moderately resistant where mortality is between 90 and 98%, and resistant where mortality was < 90% [19]. Figures were prepared using GraphPad Prism 7.02 (GraphPad Inc., La Jolla, CA, USA).

### Estimation of resistance intensity

To establish strength of pyrethroid resistance, bioassays were conducted with 5× and 10× of the discriminating concentrations of pyrethroids. Four replicates of 20–25 N’djamena F_2_ *An. coluzzii* females were exposed to 0.05, 0.25 and 0.5% of deltamethrin for 1 h. For Massakory, F_2_ females were exposed to 0.75, 3.75 and 7.5% of permethrin for 1 h. Protocols were as described in the conventional bioassays above, except for variation in the insecticides concentration [19]. Papers were prepared by the Liverpool Insecticide Testing Establishment (LITE, United Kingdom) by dissolving appropriate concentration of insecticide in acetone, and reconstituted with Dow Corning Oil, as carrier.

### Cone bioassay

To establish the efficacy of insecticide-treated nets, cone bioassays were conducted following the WHO protocol [21] using 3–4 days old F_1_ *An. coluzzii* females from N’djamena. Five replicates of ten mosquitoes were placed in plastic cone attached to four fresh, unused insecticide-containing bed nets: the Olyset®Net (containing 2% permethrin), Olyset®Plus (2% permethrin combined with 1% of the synergist, piperonyl butoxide, PBO), PermaNet®2.0 (containing 1.4–1.8 g/kg ± 25% deltamethrin), PermaNet®3.0 side panel (containing 2.1–2.8 g/kg ± 25% deltamethrin), and PermaNet®3.0 roof (4.0 g/kg ± 25% deltamethrin, combined with 25 g/kg ± 25% of PBO)]. The PermaNet and OlysetNet nets were respectively provided by Vestergaard, Lausanne, Switzerland and Sumitomo Chemical Plc, London, UK. For each experiment the five replicate tests were from five pieces cut from five different nets of the same brand. For control, three replicates of ten mosquitoes were exposed to an untreated net. Mosquitoes were exposed for 3 min, transferred immediately to paper cups and supplied with 10% sucrose. Mortality was recorded at 24 h after exposure.

### Synergist bioassay

To investigate the role of detoxification enzyme systems in the pyrethroid resistance, synergist bioassays were done using 4% PBO [an inhibitor of CYP450s [22]] against permethrin and deltamethrin. The pyrethroids and PBO (reference: WHO/VBC/81.806) were sourced from the WHO/Vector Control Research Unit (VCRU) of University of Sains Malaysia (Penang, Malaysia). Four replicates of 2–4 days old F_1_ females [20, 22–26] from both N’djamena and Massakory were pre-exposed to PBO for 1 h and then transferred to tubes containing either permethrin or deltamethrin for 1 h [19]. Mosquitoes were treated as in the WHO bioassays described above and mortalities scored after 24 h. For each experiment 25 females exposed to PBO only were used as control.

### Polymorphism analysis of the voltage-gated sodium channel

#### Genotyping of L1014F and L1014S kdr mutations

To assess the frequency of the *kdr* mutations in the field 61 F_0_ females from N’djamena and 59 females from Massakory were genotyped for the 1014F *kdr* mutation. This was done using TaqMan real-time PCR thermocycler (Agilent Mx3005) following established protocols [23, 24]. In addition, the 1014S *kdr* mutation was also genotyped using 56 females from N’djamena and 51 females from Massakory. The primers *kdr*_F (5′- CATTTTTCTTGGCCACTGTAGTGAT-3′) and *kdr*_R (5′-CGATCTTGGTCCATGTTAATTTGCA-) were used without modification. Initially, 9 μl made of 5 μl of Sensimix (Bioline), 0.25 μl of 40× Probe Mix coupled to allelic-specific primers and 4.25 μl of ddH_2_0 were mixed. 1 μl of genomic DNA (extracted from individual mosquitoes using Livak method [15]) was added to a total volume of 10 μl. Thermocycling was carried out using the following condition: initial denaturation of 10 min at 95 °C, followed by 40 cycles each of 92 °C for 15 s, and 60 °C for 1 min. Two probes labelled with fluorochromes FAM and HEX were used to detect the mutant alleles and the wild type susceptible alleles, respectively. FAM to detect the resistant allele for 1014F *kdr* (5′-ACGACAAAATTTC-3′) or (5′-ACGACTGAATTTC-3′) for 1014S *kdr*], and HEX (5′-CTTACGACTAAATTTC-3′) to detect the susceptible allele. Genotypes were scored from scatter plots of results produced by the Mx3005 v4.10 software (Agilent, Santa Clara, CA, USA). Three positive samples of known genotypes: (i) homozygote resistant for 1014F or 1014S *kdr*; (ii) heterozygote for 1014F or 1014S *kdr*; and (iii) susceptible L1014 were used as positive controls for each of the two experiments. 1 μl of ddH_2_O was incorporated into the negative control well. Correlation between the *kdr* genotype and resistance phenotype was not assessed due to the high resistance (low number of dead females).

#### Assessment of genetic diversity in the kdr locus of the voltage-gated sodium channel

To assess the strength of selection pressure acting on the *Anopheles* population, the genetic diversity of a fragment spanning exon-20 of the VGSC (starting from intron-1 of the IIS6 to intron-2) was amplified from 26 F_0_ *An. coluzzii* females (12 from N’djamena and 14 from Massakory). This fragment (Additional file 2: Figure S1) encompasses the 1014 codon where the 1014F/S mutations responsible for pyrethroids/DDT knockdown resistance is found in *An. gambiae* [25]. DNA was extracted using the Livak method and amplification carried out with the following primers described by Pinto [26]: *kdr*CL-F (5′-AAATGTCTCGCCCAAATCAG-3′) and *kdr*CL-R (5′-GCACCTGCAAAACAATGTCA-3′). A 12.5 μl mix comprise of 2x AccuStartII PCR SuperMix, containing optimised concentrations of MgCl_2_ and dNTPs (QuantaBio, Beverly, Massachusetts, USA), 0.2 μmol/L each of the forward and reverse primer was prepared. 1 μl gDNA extracted from individual female mosquitoes was added, followed by 10.5 μl of ddH_2_0 to produce a total volume of 25 μl. Amplification was carried out using the following condition: initial denaturation of one cycle at 94 °C for 3 min; followed by 35 cycles each of 94 °C for 30 s (denaturation), 60 °C for 30 s (annealing), and extension at 72 °C for 1 min; and one cycle at 72 °C for 5 min (elongation). PCR products were cleaned individually with QIAquick® PCR Purification Kit (QIAGEN, Hilden, Germany) and sequenced on both strands, using the above primers.

Polymorphisms were detected through manual examination of sequence traces using BioEdit version 7.2.3.0 (http://www.mbio.ncsu.edu/BioEdit/bioedit.html) [27] and analyses of genetic parameters of polymorphism done using the DnaSP 5.10 [28]. Different sequences were compared by constructing a maximum likelihood phylogenetic tree using MEGA 6.0 [29]. To estimate genealogies between sequences haplotype network was created with the TCS (http://darwin.uvigo.es/software/tcs.html) and tscBU [30]. All DNA sequences from the alive and dead females were submitted to the GenBank and accession numbers obtained.

### Genotyping of G119S *acetylcholinesterase-1* mutation

To detect the G119S ace-1^R^ mutation implicated in carbamate and organophosphate resistance [24] 10 bendiocarb-alive and 10 dead females from Massakory were genotyped. TaqMan assay protocol was as described for detection of the insensitive acetylcholinesterase (iAChE) [31]. 10 μl comprise of 1× Sensimix (Bioline), 80× primer/probe mix and 1 μl DNA were prepared for each sample. The probes were labelled with specific fluorophores: FAM to detect the mutant allele (S119), and HEX, to detect the susceptible allele (G119). Assay was performed using Agilent MX3005 real-time PCR machine with cycling conditions of 95 °C for 10 min, followed by 40 cycles each of 95 °C for 15 s and 60 °C for 1 min. In addition, four controls were used: (i) DNA from fully susceptible female *An. coluzzii* (Ngoussou colony); (ii) DNA from fully susceptible *An. gambiae* s.s. female (Kisumu colony); (iii) DNA from a susceptible female (SS-*ace-1*) of Central African Republic Origin [32]; and a negative control (1 μl of ddH_2_O).

### Data analysis

The results of bioassays were interpreted based as continuous variables with normal distributions and percentage mortalities ± standard error of mean (SEM) calculated based on the WHO protocol [19]. Results of mortalities from synergist-pyrethroid exposure were compared with values obtained from exposure to pyrethroid alone using a two-tailed Chi-Square test of independence, with level of significance set as *P* < 0.05, as implemented in GraphPad Prism 7.02 (GraphPad Inc., La Jolla, CA, USA). For polymorphism analysis of the fragment of the voltage-gated sodium channel allele frequency was calculated using the formula f(R) = (2 × RR + RS)/2 *N* for individuals carrying the *kdr* mutation, and f(S) = 1-f(R) for the susceptible individuals; where RR = total number of homozygote resistant; RS = total number heterozygote resistant; *N*, total number of individuals investigated. Genotype frequency was calculated as relative frequencies of the homozygote resistant and heterozygote resistant individuals.

## Results

### Composition of the mosquito species

In N’djamena 581 mosquitoes were caught indoor, out of which 539 were *An. gambiae* s.l. (18♂, 521 [443 blood fed and 78 unfed]) and 42 were of *Culex* species (26♀ bloodfed and 16 ♂). All the *Anopheles* from N’djamena were established to be *An. coluzzii*. 369♀ laid eggs and 240 of the eggs hatched successfully. From Massakory, a total of 295 mosquitoes were caught indoor, 240 of them *An. coluzzii* 89♂, (151♀ [134 bloodfed and 17 unfed]), 13 bloodfed female *An. rufipes*, three bloodfed female *An. pharoensis*, and 39 *Culex.* 143♀ of the *An. coluzzii* laid eggs and 107 egg batches hatched successfully.

### *Plasmodium* sporozoite infection

Heads/thoraces from 147♀ *An. coluzzii* (82 from N’djamena and 65 from Massakory) that laid eggs were used to detect infection with *Plasmodium*. Using both TaqMan assay and nested PCR, no any female was found infected with *Plasmodium*.

### Insecticide resistance profile of *An. coluzzii* populations

**Fig. 2:**
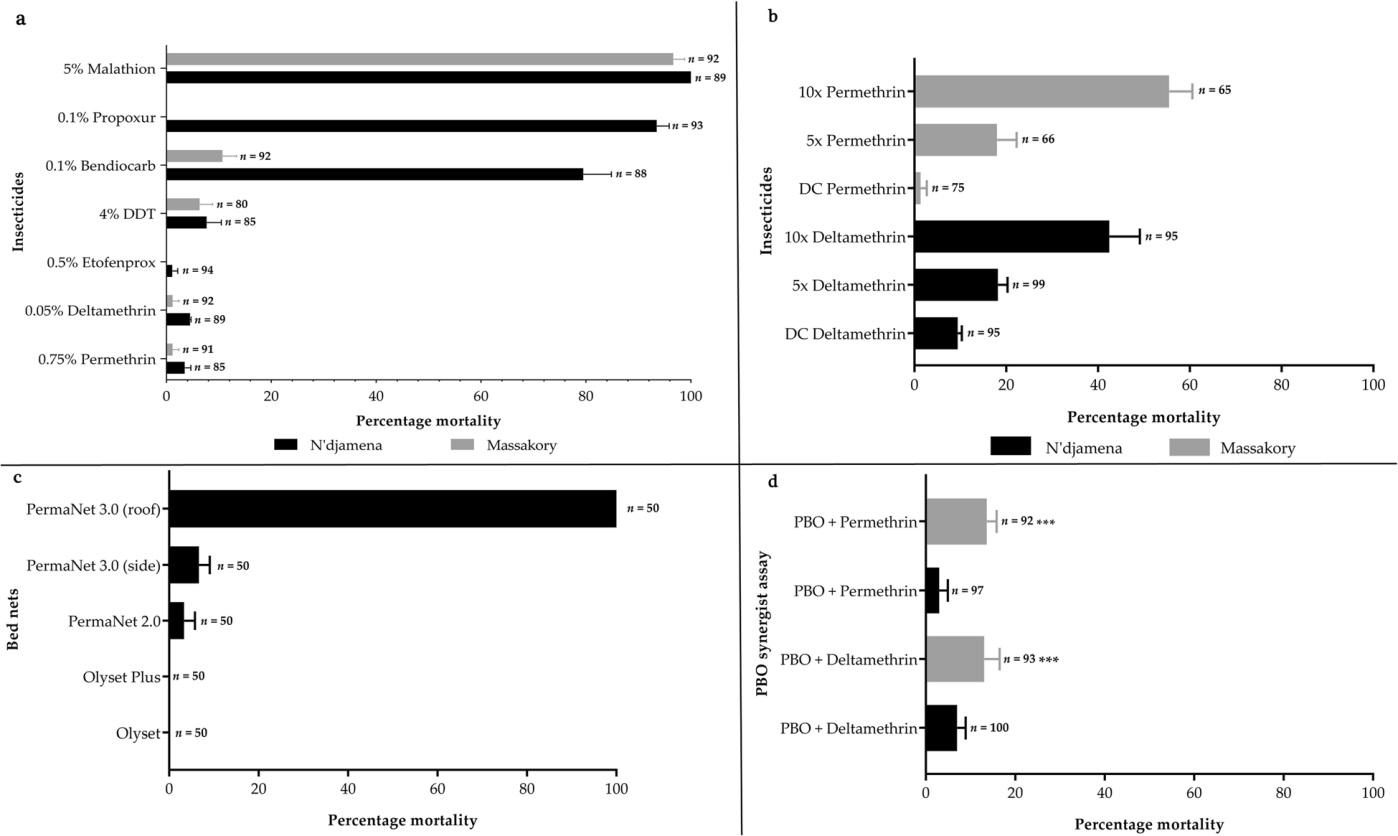
Resistance profiles of *Anopheles coluzzii* populations from N’djamena and Massakory. **a.** Results of WHO susceptibility bioassays with insecticides from different classes. Results are average of percentage mortalities from four replicates each ± SEM; **b.** Determination of resistance intensity with 5× and 10× the diagnostic concentrations of permethrin and deltamethrin. Results are average of percentage mortalities ± SEM; **c.** Results of cone bioassays with PermaNet®3.0 (side and roof), PermaNet®2.0, Olyset®Plus and Olyset®Net. Results are average of percentage mortalities ± SEM of five replicates**; d.** Effect of pre-exposure to synergist PBO against permethrin and deltamethrin. Results are average of percentage mortalities from four replicates each ± SEM. *** = statistically significant at *P* < 0.001, in a Chi-square test of independence between results from synergists bioassay and conventional bioassays

A high pyrethroid resistance was observed with mortalities of 3.49% (95% *CI*: 1.2–5.8) for permethrin in N’djamena population, and 1.19% (95% *CI:* − 1.14–3.5) from Massakory (Fig. 2a). Same pattern was observed for deltamethrin with mortalities of only 4.5% (95% *CI*: 4.3–4.7) in N’djamena and 1.19% (95% *CI:* − 1.1–3.5) for Massakory. Low mortality (1.1, 95% *CI:* − 1–3.1) was also exhibited by the N’djamena population tested with the pseudo-pyrethroid, etofenprox. Low mortalities were also obtained with DDT, at 7.7% (95% *CI*: 2.1–13.6) for N’djamena population, and 6.4% (95% *CI*: 1.5–11.2) for Massakory. A contrasting pattern between the two populations was observed with respect to bendiocarb with a moderate resistance observed in N’djamena (mortality = 79.5, 95% *CI*: 69.0–89.9), but an unusually high resistance observed in Massakory (mortality = 10.7, 95% *CI*: 5.5–15.9). Propoxur, was only tested with the N’djamena population with a mortality of 93.5% (95% *CI*: 87.8–99.2) obtained. Both populations were susceptible to malathion, ranging from 100% mortalities 95% *CI*: 100–100) from N’djamena population, to 96.6% (95% *CI*: 92.4–100.9) obtained from Massakory. Full susceptibility (100% mortalities with all insecticides) was obtained with the Ngoussou colony.

### Estimated resistance intensity

To establish the intensity of resistance bioassays were conducted with the 5x and 10x the diagnostic doses of deltamethrin for N’djamena F_2_ population and permethrin for the Massakory F_2_ population. High intensity resistance was observed in the populations from both sites (Fig. 2b). A mortality of only 18.2% (95% *CI*: 14.1–22.3) was obtained with N’djamena population when exposed to 5× deltamethrin compared to 9.5% (95% *CI*: 7.7–11.2) for 1× deltamethrin. Mortality increased to 42.6% (95% *CI*: 29.5–55.6) for 10× deltamethrin. For Massakory, a repeat of bioassays with 1x permethrin (discriminating concentration) established a mortality of only 1.3% (95% *CI*: − 1.3–3.9). This increased to 18.0% (95% *CI*: 9.7–26.3) with 5× permethrin and only 55.5% (95% *CI*: 45.5–65.4) with 10× permethrin.

### Test of bet net efficacy using cone bioassay

To evaluate the efficacy of commercially available treated bed nets cone bioassays were conducted with the N’djamena population. A complete loss of efficacy was observed with the pyrethroid based Olyset®Net (no mortality at all) and PermaNet®2.0 (mortality = 3.3, 95% *CI*: 1.2–9.9) (Fig. 2c). Low mortality was obtained from mosquitoes exposed to side panel of PermaNet®3.0 (mortality = 6.7, 95% *CI*: 0.1–13.2). Also, no mortality was obtained from exposure to Olyset®Plus containing PBO. In contrast, 100% mortality was seen from exposure to the roof of PermaNet®3.0 (containing PBO). No mortality was obtained with the control populations exposed to untreated bed nets. Full susceptibility (a mortality of 100%) was obtained with Ngoussou colony with PermaNet®2.0, and a high mortality of 88% ± 3.7% with Olyset®Net.

### Investigating the potential role of metabolic resistance using synergist bioassays

Pre-exposure to PBO recovered some susceptibility to both permethrin and deltamethrin, especially in the Massakory populations (Fig. 2d). For N’djamena no significant increase in mortalities was observed, respectively for permethrin and deltamethrin when comparing results of conventional bioassay without PBO (3.5 and 4.5%) respectively, to results of synergized bioassay with PBO (3.0% [95% *CI*: 0.5–8.5, *χ*^2^ = 0.03, df = 1, *P* = 0.86] and 7.0% [3.5–12.5, *χ*^2^ = 0.54, df = 1, *P* = 0.46]). Slightly higher synergistic effect was observed in Massakory population, with mortalities increasing for permethrin from 1.2% without PBO to 13.6% (95% *CI*: 11.7–22.9, *χ*^2^ = 11.66, df = 1, *P* = 0.0006) after pre-exposure to PBO. Similarly, for deltamethrin an increase in mortality was also observed, from 1.2 to 13.0% (95% *CI*: 4.0–23.1, *χ*^2^ = 8.76, df = 1, *P* < 0.00031) after PBO pre-exposure. This suggests the possible role of cytochrome P450s in the resistance observed resistance. No mortality was observed in all controls.

### Genotyping of the *kdr* mutations and polymorphism analysis of the voltage-gated sodium channel

#### The presence of 1014F and 1014S kdr mutations in the field

Both the 1014F and 1014S *kdr* mutations were detected in Massakory and N’djamena (Table 1). Overall, the frequency of the 1014F *kdr* mutation was slightly higher in Massakory (64%), compared to N’djamena (57%). There is also differences in the genotype distributions, for example, the Massakory homozygote resistant individuals (1014F/F) exhibited higher frequencies compared with heterozygotes (1014 L/F). In contrast, in N’djamena the heterozygote resistant individuals have higher frequencies compared with the homozygote resistant. In contrast, the 1014S *kdr* was found only at heterozygote state with a very low frequency of less than 20% in both locations.

**Table 1:** Genotype and allele frequencies of 1014F and 1014S *kdr* mutations in *An. coluzzii* populations

Population	Genotype					Allele	
1014F *kdr*							
	RR (%)	RS (%)	SS (%)	Total	2 N	f(R)	f(S)
N’djamena	17 (27.9)	31 (50.8)	11 (18.0)	61	122	0.57	0.43
Massakory	28 (47.5)	19 (32.2)	12 (20.3)	59	118	0.64	0.36
1014S *kdr*							
N’djamena	0 (0)	10 (17.9)	46 (82.1)	56	112	0.089	0.91
Massakory	0 (0)	8 (15.7)	43 (84.3)	51	102	0.078	0.92

RR, homozygous resistant, RS, heterozygous resistant, SS, homozygous susceptible. In total, 120 mosquitoes were genotyped for the 1014F *kdr* mutation, and 107 for the 1014S *kdr*. f(R): frequency of resistance allele; f(S): frequency of susceptible allele

No mosquito carrying both 1014F and 1014S *kdr* resistant allele was detected. Correlation between the *kdr* genotype and resistance phenotype was not assessed due to low number of dead females.

#### Genetic diversity and phylogenetic analysis of fragment of voltage-gated sodium channel

**Fig. 3:**
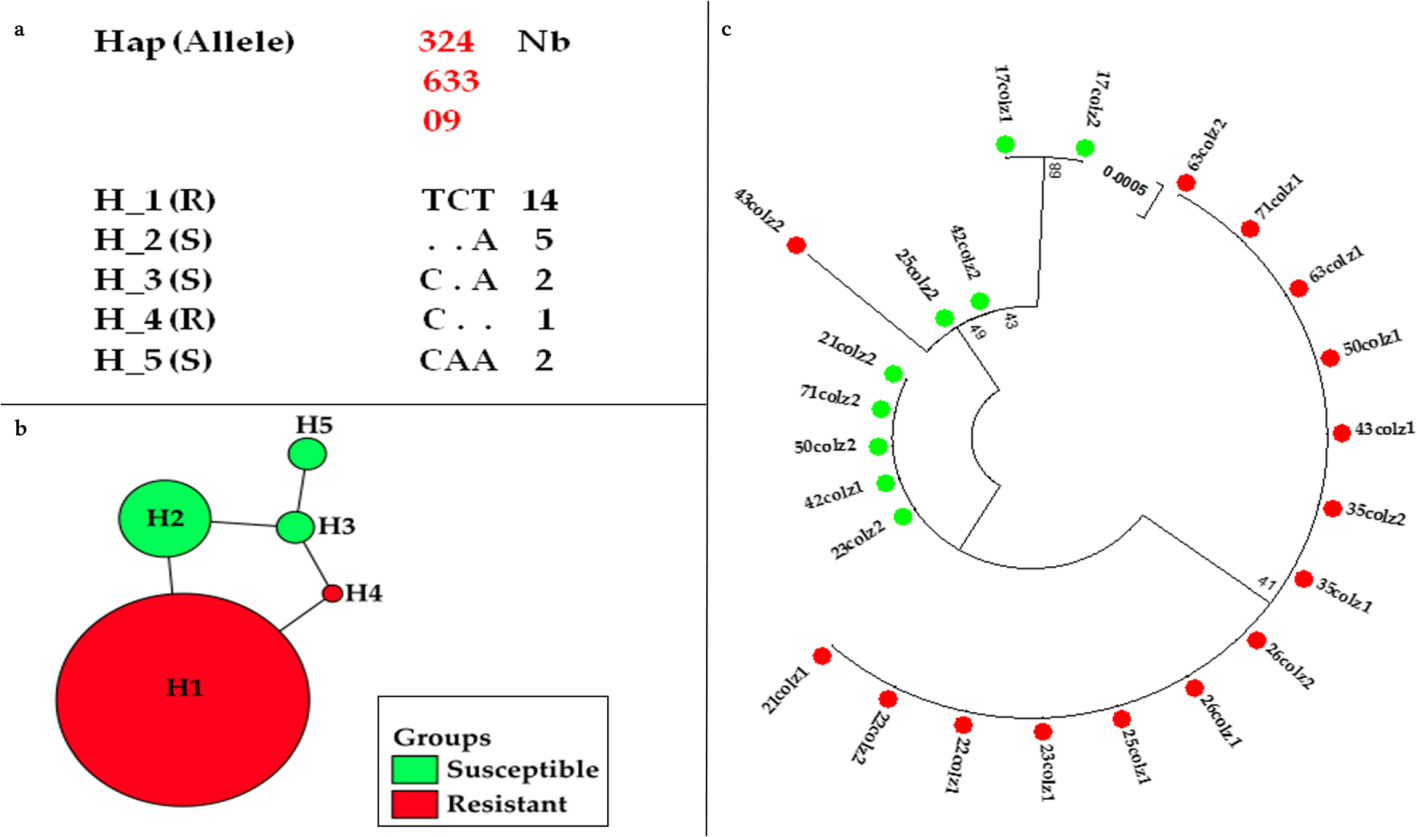
Genetic diversity of a fragment of the *VGSC* spanning exon 20 in *Anopheles coluzzii* from N’djamena. **a.** Analysis of polymorphism of 494 bp fragments of the *VGSC*; **b.** TCS and tcsBU haplotype network showing low polymorphism in exon 20; **c.** Phylogenetic tree of the *VGSC* sequences. Green dots represent the susceptible haplotypes, red dots are the resistant haplotypes

A 494 bp fragment spanning the 1014 codon was sequenced for 12 individuals from N’djamena and 14 from Massakory. The sequences from N’djamena produced five distinct haplotypes, with low polymorphism (S = 3), and haplotype diversity of 0.63 (Table 2). Two of these haplotypes, H_1 (the predominant haplotype) and H_4 had the 1014F allele at frequencies of 58.3% (14/24 sequences) and 4.2% (1/24), respectively. The three remaining haplotypes H_2, H_3 and H_5 had 1014 L susceptible allele, at frequencies of 20.8% (5/24), 8.3% (2/24) and 8.3% (2/24), respectively (Fig. 3a, −b). The haplotypes cluster on a maximum likelihood phylogenetic tree according to their genotype with the ones containing the 1014F codon separate from those harboring the 1014 L codon (Fig. 3c). Comparison of N’djamena haplotypes with four *kdr* bearing haplotype previously detected across Africa [26] revealed that the major haplotype H_1 and the haplotype H_4 belong respectively to the H1-1014F and H3-1014F resistance haplotypes, predominant in West/Central Africa, suggesting gene flow in *An. coluzzii* population across the region. Haplotype network tree analysis showed that haplotype H_4 is separated by one mutational step from the ancestor haplotype H_1 (Fig. 3b).

**Table 2:** Summary statistics for polymorphism of the fragment of voltage-gated sodium channel haplotypes from N’djamena and Massakory *An. coluzzii* populations

Population	*n*	S	h	H_d_	Syn	Nonsyn	π	D (Tajima)	D* (Fu and Li)
N’djamena	12	3	5	0.63	2	1	0.00201	0.59^ns^	0.97 ^ns^
Massakory	14	3	4	0.56	0	1	0.0027	1.80^ns^	0.95^ns^
All	16	3	5	0.72	0	1	0.0024 (1.30)	0.94^ns^	1.36^ns^

*n*, Number of sequences; S, Number of polymorphic sites; h, Haplotype; H_d_, Haplotype diversity; Syn, Synonymous mutations; Nonsyn, Non-synonymous mutations; π, nucleotide diversity; Tajima’s D and Fu and Li’s D statistics, ns, not significant

**Fig. 4:**
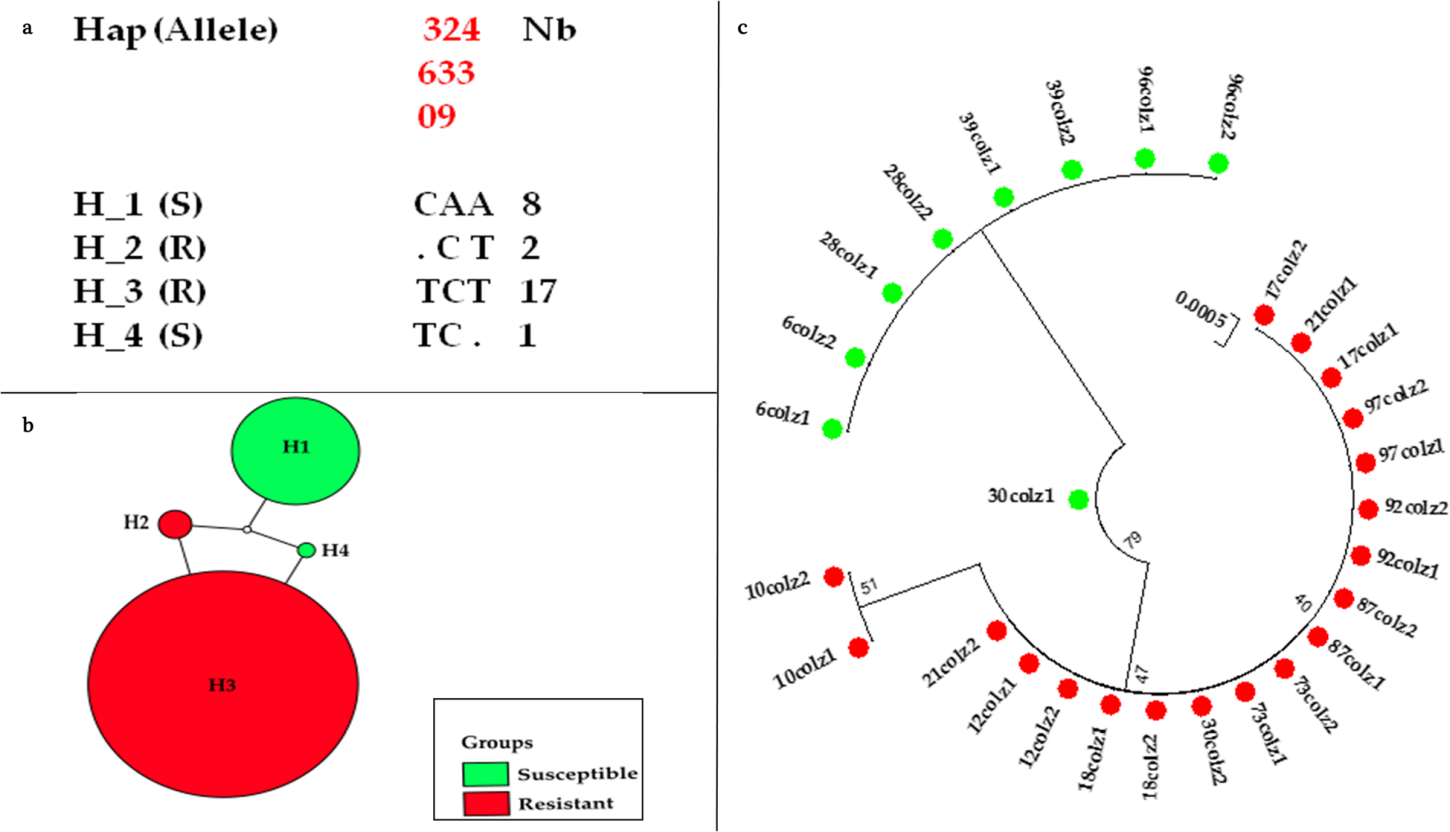
Genetic diversity of a fragment of *VGSC* spanning exon 20 of *Anopheles coluzzii* from Massakory. **a.** Analysis of polymorphism of 494 bp fragments of the *VGSC*; **b.** TCS and tcsBU haplotype network showing low polymorphism in exon 20; **c.** Phylogenetic tree of the *VGSC* sequences. Green dots represent the susceptible haplotypes, and red dots are the resistant haplotypes

The 14 sequences from Massakory produced four haplotypes, with low polymorphism (S = 3), and lower haplotype diversity (0.56) compared with N’djamena (Table 2). Two haplotypes H_2 and H_3 contained the 1014F allele, while haplotypes H_1 and H_4 harbored the 1014 L susceptible allele. Haplotype H_3 is the major haplotype constituting 60.7% (17/28 sequences) (Fig. 4a and -b). The haplotype H_1 is the second major haplotype containing 1014 L susceptible allele at frequency of 28.6% (8/28). This reduced number of haplotypes suggests a restricted polymorphism of the VGSC in link with the near fixation of the 1014F in this population. Comparison of the Massakory haplotypes with four *kdr* bearing haplotype previously detected across Africa revealed that the major haplotype H_3 and resistance haplotype H_2 belong respectively to the H1-1014F and H3-1014F resistance haplotypes, predominant in West/Central Africa [26].

### Presence of G119S *acetylcholinesterase-1* mutation

To investigate the underlying target-site resistance mechanism responsible for high bendiocarb resistance as observed in Massakory population, 10 bendiocarb-alive and 10 dead females, randomly selected were genotyped for the G119S *ace-1* mutation. All samples genotyped were homozygote susceptible (119G), suggesting that the bendiocarb resistance could be because of metabolic mechanism.

## Discussion

Any push for pre-elimination of malaria in the Sahel, notably in Chad will require a better knowledge of the malaria vectors in this region. Effective control of malaria also relies heavily on routine collection of local malaria vectors, establishing their role in transmission and characterisation of their resistance status. Such information is scanty in the Sahel region of Chad Republic. To provide data which could feed into decision making by the Chadian National Malaria Control Program, the major malaria vector was established in two sites at the Sahel region of Central Chad. The role of the dominant vector *An. coluzzii* in malaria transmission was investigated, its resistance to the major public health insecticides profiled, and the possible molecular mechanisms driving the resistance in the field characterized.

The finding of *An. coluzzii* as the major malaria vector in N’djamena and Massakory is not surprising, for this vector has been of recent found to constitute 94.9% *of Anopheles* collected indoor at the Sahel of Douiguia, in Chad [33], and ~ 98% of collection from Kome, in southern Chad [8]. Indeed, *An. coluzzii* had been established of recent as the major malaria vector in neighbouring regions sharing similar ecological characteristics, e.g. in Sahel/Sudan savannah of northern Nigeria [34], and in Sahel of Niger Republic [35]. This is in contrast to the previous observations/predictions of *An. arabiensis* as the predominant vector in Chad, e.g. in N’djamena and Mandelia [10], and in Bongor, Guelendeng and Kélo [11]. *Anopheles coluzzii* is progressively becoming the dominant vector species in the Sahel compared to the *An. arabiensis* and *An. gambiae* s.s. which are hard to come by [21, 36]; although longitudinal studies will help to further establish this trend for the location studied here. In recent years *An. coluzzii* has possibly adapted well in drier, semi-arid regions, as it was reported more than the other two sibling species in various studies from the Sudan/Sahel of the neighbouring countries, e.g. in Mali [37] in northern Nigeria [38], and in northern Chad [33]. This species is thought to have higher breeding sites exploiting capabilities, associated with anthropogenic activities, such as farming in rainy season, and irrigation, which create semi-permanent and permanent breeding sites [39].

Despite the high number of bloodfed *An. coluzzii* females collected in both N’djamena and Massakory, no single female was found infected with *Plasmodium*. This is in contrast to previous studies from Chad, for example, a sporozoite index of 4.5% was recently reported in *An. coluzzii* from Douiguia [33] and in 2010 the sporozoite rate of 2.5% were found in N’djamena [9]. The absence of *Plasmodium* infected females observed in this study is possibly due to suppression of malaria transmission from implementation of seasonal malaria chemoprevention (SMC) (https://www.malariaconsortium.org/), as part of 2014–2018 Strategic Plan of Chad’s National Malaria Control Program (NMCP). This campaign covered the three regions of Chad, including N’djamena and Massakory. However, one of the limitations of this study is that the role of the few secondary vectors (e.g. *An. rupifes* and *An. pharoensis*) in malaria transmission was not investigated, due to small sample size.

As observed in neighbouring Sahel regions sharing similar ecological characteristics, e.g. in Nigeria [34] and Niger [35], insecticide resistance has also escalated in *An. coluzzii* populations from Chad. Studies conducted at N’djamena between 2006 and 2008 had reported mortalities of 71% for permethrin, 82% for deltamethrin and up to 98% for DDT [9]. By 2014 resistance had increased with mortalities falling to just 2% for permethrin and deltamethrin, while a dramatic drop in DDT susceptibility was observed from 98% at 2010 to 0% in 2014 [9]. This pattern is in line with our findings of a very low mortalities with permethrin, deltamethrin and DDT. DDT resistance was first observed at the cotton-growing Savannah of Donia, at southern Chad where *An. coluzzii* was consistently found [9, 10]. The rise of DDT resistance in the semi-arid central Chad coincide with the recent population expansion of *An. coluzzii* in this region. The intense pyrethroid resistance is reflected in the findings of the resistance intensity bioassays, with the two populations tested showing high resistance to even 10× the discriminating concentrations of permethrin and deltamethrin. The high pyrethroid resistance was also evident in the loss of efficacy of insecticide-treated bed nets, e.g. PermaNet®2.0, PermaNet®3.0 (the side panel containing deltamethrin only), and Olyset Net. These findings are like recent observation in *An. coluzzii* population from savannah of central Cameroon [40]. The observation of recovery of susceptibility from exposure to PermaNet®3.0 (the roof containing PBO, in addition to deltamethrin) agrees with the findings from synergist bioassays from which statistically significant recovery of pyrethroid-induced mortalities were obtained in Massakory populations, following pre-exposure to PBO. However, the loss of activity with Olyset®Plus shows that this resistance escalation could also impact some PBO-based nets. Indeed, loss of activity of Olyset®Plus nets have been described in several studies with *An. coluzzii* [40, 41].

Initial studies on presence of the *kdr* mutation in *Anopheles gambiae* s.l. from Kélo, southwest of Chad revealed presence of the 1014F *kdr* mutation in the S molecular form (*An. gambiae* s.s.), and its absence in the M form (*An. coluzzii*) and *An. arabiensis* [11]. Indeed, *kdr* genotyping carried out by Foster and colleagues [9] from collection in 2008 revealed presence of both 1014F and 1014S *kdr* mutations in only *An. gambiae* s.s. collected from far south, in the humid region of Chad. The *kdr* mutations were absent in *An. arabiensis* (the major malaria vector in N’djamena [42]) and the *An. coluzzii* collected in N’djamena, Bongor and Donia. However, by 2013 *An. coluzzii* collected at Kome, southern Chad exhibited a high frequency of 1014F *kdr* mutation (54.9%) compared to the *An. gambiae* s.s. and *An. arabiensis* from same locality [8]. The frequencies of 1014F *kdr* mutations found in this study are at comparable to the findings of Dadzie and colleague [8], and meant in some 5 years the frequency of the 1014F *kdr* mutation has remained the same. This, together with the recovery of mortality from PBO pre-exposure suggests a major role of metabolic resistance mechanisms, which were not explored through genome-wide transcriptional analyses, in this study. The low frequency of the 1014S *kdr* mutation at both sites and the absence of homozygote resistant individuals suggests possible fitness cost associated with the homozygosity of this mutation. The rise of the *kdr* mutation in *An. coluzzii* is probably due to the comparably higher selective pressure this species is subjected to, as a result of exposure to agrochemicals, as it adapts to survive year round in areas with extensive human activities [8, 39]. Unfortunately, genotype-phenotype association was not established due to a very low number of dead females from both sites.

The low haplotype and nucleotide diversity seen in the partial fragment of the VGSC from N’djamena and Massakory suggests a reduced polymorphism in the sodium channel. The findings of the major resistance haplotypes for both 1014F and 1014S *kdr* mutation matching the predominant haplotypes associated with resistance across Central Africa [32, 43] suggests a gene flow in *An. coluzzii* across the region.

During 2008–2010 a full susceptibility to carbamates and organophosphates was documented in *An. coluzzii* populations in Chad. For example, in Guelendeng, not far from N’djamena [11], in Mandelia, Bongor and Donia [10]. The findings of high bendiocarb resistance in *An. coluzzii* from Massakory is consistent with the observation of Dadzie in 2016 [8], where they reported mortalities of only ~ 20% in *An. coluzzii* population from Kome, southern Chad. The bendiocarb resistance in Massakory population is higher than recently observed in the Sahel of Nigeria and Niger where *An. coluzzii* populations exhibited moderate bendiocarb resistance [34, 35]. It is possible that this carbamate resistance was selected by agricultural use of carbamate-based pesticides, as no IRS has been implemented in Chad with this class of insecticides.

The absence of the G119S *ace-1* mutation in the highly bendiocarb-resistant Massakory populations correlates with the phenotypic susceptibility to organophosphates. This confirms no cross resistance and point to possible metabolic mechanisms associated with the bendiocarb resistance. Indeed, this mutation has been reported as absent in *An. gambiae* s.l. populations on several occasions from studies carried out across Chad, using both susceptible populations [9] and the resistant ones [8]. However, with only 10 females each of alive and dead used to access presence of this mutation, presence of this mutation at a low frequency cannot be ruled out.

The major limitations of this study are: (i) that the role of the few secondary vectors collected (e.g. *An. rupifes* and *An. pharoensis*) in malaria transmission was not investigated, due to small sample size; (ii) contributions of metabolic resistance mechanism, by the major detoxification enzymes such as the cytochrome P450s were not explored using the genome-wide transcriptional analyses; and (iii) only 10 females each of bendiocarb-alive and dead were used to determine presence of G119S target-site mutation. Presence of this mutation at a low frequency cannot be ruled out.

## Conclusions

This study finds disproportionately high pyrethroid resistance in the major malaria vector *An. coluzzii* from Chad, which will pose serious threat to malaria control using bed nets. The unusually high carbamate resistance observed in the field populations of this vector may affect the future control measures in Chad, using the carbamate-based indoor residual spraying. However, PBO-containing combination bed net PermaNet®3.0 was found to be still effective in killing this species; thus, control measures should include distribution of this class of bed net. The finding of full susceptibility to organophosphates make them important alternatives for indoor residual spraying, which could help in pre-elimination of malaria in the Sahel of Chad.

## Supplementary information

**Additional file 1:** Multilingual abstracts in the five official working languages of the United Nations.

**Additional file 2: Figure S1.** The nucleotide sequences of the voltage-gated sodium channel fragment, spanning the *kdr* locus.

## Data Availability

DNA sequences reported in this paper were deposited at GenBank (Accession No. MN031997-MN032022).

## Abbreviations

ace-1: acetylcholinesterase-1
CI: confidence interval
CRID: Centre for Research in Infectious Diseases
CYP450s: Cytochrome P450s
ddH_2_0: double distilled water
DDT: dichlorodiphenyltrichloroethane
DNA: deoxyribonucleic acid
gDNA: genomic DNA
IRS: indoor residual spraying
kdr: knockdown resistance
LLINs: long-lasting insecticidal nets
ml: millilitre
mm: millimetre
NMCP: National Malaria Control Program
PBO: piperonylbutoxide
s.l.: sensu lato
s.s.: sensu stricto
SEM: standard error of mean
SMCP: seasonal malaria chemoprevention
VCRU: Vector Control Research Unit
VGSC: voltage-gated sodium channel
WHO: World Health Organization
