# Supplementary material for: High insecticide resistance in the major malaria vector Anopheles coluzzii in Chad Republic
Source: Infect Dis Poverty. 2019 Dec 3;8:100. doi: 10.1186/s40249-019-0605-x (PMC6892245; doi:10.1186/s40249-019-0605-x)
Supplement: Supplementary file 1 — Additional file 1: Multilingual abstracts in the five official working languages of the United Nations. [file 40249_2019_605_MOESM1_ESM.pdf]

Translation of the abstract into the five official working languages of the United Nations

مقاومة عالية ضد المبيدات الحشرية فى ناقلات الملاريا الرئيسية بعوض الأنوفيليس فى جمهورية تشاد

سليمان س. إبراهيم، أمين ن. فاضل ، ماجيلان تشواكوى ، إباى تيرينس ، موريل ج. وندجى ، ميكاريم تشوبو ، كليمينت كيرا-هينزومبى ، ساموبل ونجى، و تشارلز س. وندجى

ملخص

المعلومات الأساسية:تعتبر منطقة الساحل في جمهورية تشاد مرشحاً رئيسياً لمرحلة ما قبل القضاء على الملاريا. لتيسير الجهود المبذولة فى مرحلة ما قبل القضاء على الملاريا فى هذه المنطقة ، تم توصيف اثنين من تجمعات الأنوفيلة الغامبية من وسط جمهورية تشاد ، وطرق مقاومتها للمبيدات الحشرية والآليات الجزيئية المحتملة التى تحفز المقاومة فى الميدان الذى تم بحثه.

الطرق: تم جمع أنثى أنوفيلة غامبية متغذية على الدماء s.l. تمكث بمكان مغلق ، من نجامينا و ماساكورى، تشاد فى 2018 وتم توصيفها تبعاً للأنواع المكونة ، وتم تحديد معدل الإصابة باستخدام تحليل تاكمان TaqMan. تم تقدير قابلية التعرض لمختلف المبيدات الحشرية عن طريق فحوصات الأنبوب الحيوية لمنظمة الصحة العالمية. أجريت فحوصات المخروط الحيوية باستخدام ناموسيات معالجة بمبيدات حشرية طويلة الأجل (LLINs). وتم تحليل النتائج باستخدام اختبار مربع كاي. تم فحص علامات مقاومة الصدمة (kdr) و 1-aceبواسطة التنميط الجينى لتاكمان TaqMan.

النتائج: كانت الأنوفيلة الغامبية هي ناقلات المرض الرئيسية التى وجدت فى ناجامينا (100%) وماساكورى (~94%). لم يتم العثور على بلازمودات فى 147 أنثى 0 أنوفيلة غامبية متغذية على الدماء (82 من ناجامينا و 65 من ماساكورى). وقد لوحظت مقاومة عالية للبيروثرويد بمعدل وفيات أقل من 2٪ بالنسبة للبيرميثرين ، والدلاميثرين ، والإيتوفينبروكس; ومع وفيات أقل من 50٪ و 60٪ بعد التعرض لجرعات تشخيصية قدرها 10 × من الدلتاميثرين والبيرميثرين على التوالي. لكلا الموقعين ، تم ملاحظة وفيات أقل من 10% بالنسبة للدى دى تى . أعادت الاختبارات الحيوية التآزرية بواسطة البيرونيل بيوتوكسايد البريثرويد بشكل ملحوظ فى تجمعات ماساكورى ، متضمنة CYP450s (معدل الوفيات = 13.6 ٪ بالنسبة للبيرميثرين ، 22.8 = χ 2، f = 1d ، P = 0.0006 ؛ معدل الوفيات = 13.0٪ بالنسبة للدلتاميثرين ، 8.8 = χ 2 ، df = 1 ، P <0.00031). أثبتت اختبارات المخروط الحيوية فقداناً تاماً لفعالية الناموسيات المعالجة بمبيدات حشرية طويلة الأجل القائمة على البيرثرويد; واستعادة 100 ٪ من فعاليتها بعد التعرض لسطح PermaNet®3.0 ، الذي يحتوي على البيرونيل بيوتوكسايد. كان كلا التجمعين عرضة للإصابة بالملاثيون ، ولكن لوحظت مقاومة بنديوكارب عالية في تجمعات ماساكوري. يشير غياب طفرة 1-cea إلى دور المقاومة الأيضية في مقاومة بنديوكارب. تم العثور على طفرات F1014 و S1014 في كلا التجمعات في حوالي 60 ٪ ولأقل من 20 ٪ على التوالي. كشف تسلسل 1-intron لقناة الصوديوم الفولتية عن تنوع جيني منخفض مما يشير إلى انخفاض معدل تعدد الأشكال.

الاستنتاجات: المقاومة المتعددة في تجمعات الأنوفيلة الغامبية من تشاد تسلط الضوء على التحديات المرتبطة بنشر الناموسيات المعالجة بمبيدات حشرية طويلة الأجل وآثار الرش الموضعي في الأماكن المغلقة على ساحل هذه الدولة. قد تكون الناموسيات المعالجة بمبيدات حشرية طويلة الأجل المتآزرة للبيروثرويدات (مثلPermaNet®3.0) والرش الموضعى للأماكن المغلقة القائم على الفوسفات العضوى بدائل لمكافحة الملاريا فى هذه المنطقة.

Translated from English version into Arabic by Hager Ryan, revised by Amal Imam, through

TRANSLATORS
WITHOUT BORDERS

乍得共和国主要疟疾传播媒介 *Anopheles coluzzii* 杀虫剂抗性较高

Sulaiman S. Ibrahim, Amen N. Fadel, Magellan Tchouakui, Ebai Terence, Murielle J. Wondji, Micareme Tchoupo, Clement Kérah-Hinzoumbé, Samuel Wanji, and Charles S. Wondji

摘要

引言：乍得共和国萨赫勒地区是疟疾预消除的主要候选地区。为了促进该地区的消除工作，本研究对乍得共和国中部的两个按蚊种群进行了特征描述，研究了它们对杀虫剂的抗性以及可能的抗性分子机制。

方法：2018 年在乍得的 N’djamena 和 Massakory 采集室内静息的饱血雌性冈比亚按蚊，并对其物种组成进行了鉴定，采用 TaqMan 法测定了感染率。使用 WHO 生物测定法评估了蚊虫对各种杀虫剂的敏感性。使用各种长效杀虫蚊帐(LLINs)进行了生物测定（WHO cone bioassay）。采用卡方检验分析结果。通过 TaqMan 基因分型研究了击倒抗性(*kdr*)和 *ace-1* 标记。
结果：*Anopheles coluzzii* 是 N’djamena (100%)和 Massakory(94%)的主要媒介。147 只饱血的 F_0 *Anopheles coluzzii* 未检出疟原虫（82 只来自 N’djamena，65 只来自 Massakory)。观察到高强度拟除虫菊酯抗性，暴露于氯菊酯、溴氰菊酯和醚菊酯蚊虫死亡率< 2%，暴露于 10 倍诊断剂量的溴氰菊酯和氯菊酯的蚊虫死亡率分别为< 50%和< 60%。在这两个地区，DDT 的死亡率均低于 10%。在 Massakory 群体中，多功能氧化酶抑制剂增效醚(piperonyl butoxide)可显著恢复拟除虫菊酯敏感性，涉及 CYP450s (氯菊酯死亡率= 13.6%，χ^2 = 22.8，df = 1, P = 0.0006；溴氰菊酯死亡率= 13.0%, χ^2 = 8.8，df = 1, P < 0.00031)。Cone 生物测定法确认了拟除虫菊酯为基础的 LLINs 完全无效；暴露于含增效醚的 PermaNet®3.0 后敏感性可完全恢复。两个种群均对马拉硫磷敏感，但 Massakory 种群对恶虫威抗性较高。*ace-1* 突变的缺失说明代谢抗性在恶虫威抗性中的作用。1014F 和 1014S 的突变在两个种群中分别约占 60%和< 20%。电压门控钠通道的内含子-1 序列结果显示遗传多样性较低，表明多态性减少。
结论：来自乍得的 *An. coluzzii* 种群的多重抗性凸显了与萨赫勒地区 LLINs 部署和室内滞留喷洒有关的挑战。拟除虫菊酯-增效剂 LLINS（如 Permanet®3.0）和基于有机磷的 IRS 可能是该区域疟疾控制的替代方案。

Translated from English version into Chinese by Xin-Yu Feng, edited by Pin Yang

CHINESE CENTER FOR DISEASE CONTROL AND PREVENTION
NATIONAL INSTITUTE OF PARASITIC DISEASES

Forte résistance aux insecticides chez le principal vecteur du paludisme *Anopheles coluzzii* en République du Tchad

Sulaiman S. Ibrahim, Amen N. Fadel, Magellan Tchouakui, Ebai Terence, Murielle J. Wondji, Micareme Tchoupo, Clement Kérah-Hinzoumbé, Samuel Wanji, et Charles S. Wondji

Résumé
Contexte: La région sahélienne de la république du Tchad est un candidat de choix concernant la pré-élimination du paludisme. Pour faciliter les efforts de pré-élimination du paludisme dans cette région, deux populations de *Anopheles coluzzii* en république du Tchad ont été sélectionnées, leur profil de résistance aux insecticides ainsi que les éventuels mécanismes moléculaires véhiculant sur le terrain, ont été examinés.
Méthodes: Des moustiques femelles gorgées de sang *Anopheles gambiae* s.l. reposant à l'intérieur, ont été recueillies à N'djaména et Massakory, en république du Tchad en 2018, et analysées pour mettre en évidence les différentes espèces ; le taux d'infection a été déterminé au moyen du test TagMan. La sensibilité à des insecticides divers a été évaluée à l'aide des tests biologiques de l'OMS, au moyen de tubes. Les tests biologiques en cylindre ont été effectués par l'utilisation de moustiquaires imprégnées d'insecticide à effet durable (MIDs). Les résultats ont été analysés par le biais du test Chi Square. Les marqueurs de résistance à l'effet mortel (*kdr*) et *ace-1* ont été étudiés par génotypage Taqman.
Résultats: *Anopheles coluzzii* a été le principal vecteur du paludisme identifié à N'djaména (100%) et Massakory (~94%). Aucune espèce de *Plasmodium* n'a été identifiée chez 147 moustiques femelles gorgées de sang$_0$ *An. coluzzii* (82 en provenance de N’djamena et 65 en provenance de Massakory). Une très forte intensité de résistance au Pyréthroide a été observée avec des mortalités de < 2% au perméthrine, deltaméthrine et etofenprox, et des mortalités de < 50% et de < 60% à la suite d'une exposition d'une concentration discriminante multipliée par 10 de deltaméthrine et de perméthrine respectivement. Dans les deux villes, < 10% de mortalités ont été observées avec le DDT Les tests biologiques aux insecticides synergistes à base de pipéronylbutoxide ont fortement révélé la sensibilité au pyréthroide dans les populations de Massakory, impliquant CYP450s (mortalité = 13.6% par le perméthrine, χ^2 = 22.8, df = 1, P = 0.0006; mortalité = 13.0%

par le deltamethrine, $\chi^2 = 8.8$, df = 1, $P < 0.00031$). les tests biologiques en cylindre ont révélé une perte d'efficacité lors de l'imprégnation de pyréthroide à effet durable sur les moustiquaires ; et 100% de recouvrement de sensibilité à la suite d'une exposition de PermaNet 3.0 (sur la partie supérieure des moustiquaires) contenant du Pipéronylbutoxide. Les deux populations ont été sensibles au malathion, mais une forte résistance au bendiocarbe a été observée chez la population de Massakory. L'absence de mutation du gène *ace-1* souligne le rôle de la résistance métabolique dans la résistance du bendiocarbe. Les deux mutations 1014F et 1014S ont été identifiées chez les deux populations autour de 60% et < 20% respectivement. Le séquençage d'Intron-1 sur le canal sodique voltage-dépendant a révélé un amoindrissement de la diversité génétique ce qui laisse supposer un polymorphisme réduit.

Conclusions: Les résistances multiples chez les espèces *An. coluzzii* en République du Tchad met en lumière les difficultés liées à l'utilisation des moustiquaires imprégnées d'insecticides à effet durable et la pulvérisation d'insecticides à l'intérieur des habitations (IRS) dans la région sahélienne de ce pays. les moustiquaires imprégnées de synergistes à base de pyréthroide (e.g PermaNet 3.0) et la pulvérisation d'insecticides organophosphorés à l'intérieur des habitations peuvent être des alternatives dans la lutte du paludisme dans cette région.

Translated from English version into French by Véronique Moquet, revised by Emilie Rigault Fourcadie, through

TRANSLATORS
WITHOUT BORDERS

Высокая устойчивость к инсектицидам одного из основных разносчиков малярии в Республике Чад *Anopheles coluzzii*

Сулейман С. Ибрагим, Амен Н. Фадель, Магеллан Чуакуи, Эбай Теренс, Мюриэль Дж. Уонджи, Микарем Тчупо, Клемент Кера-Хинзумбе, Самуэль Уанджи и Чарльз С. Уонджи

Реферат

Предпосылки: регион сахеля Республики Чад является близок к ликвидации малярии. С целью оказания содействия работам по подготовке к искоренению мы охарактеризовали две популяции *Anopheles coluzzii* из центрального Чада и исследовали профиль их устойчивости к инсектицидам и возможные молекулярные механизмы, ответственные за такую устойчивость.

Методы: сидящие наполненный кровью самки *Anopheles gambiae* s.l. собирались в закрытых помещений в Нджамене и Массакори (Чад) в 2018 году и затем характеризовались по родовому составу, а доля инфицированности определялась в исследовании по TaqMan. Подверженность воздействию различных инсектицидов оценалась с использованием пробирочного биоанализа ВОЗ. Биоанализ с конусом ВОЗ проводился для различных противомоскитных сеток длительного пользования (LLINs). Результаты измерения были обработаны с помощью теста хи-квадрат. Устойчивость к нокдауну (*kdr*) и маркеры *ace-1* были исследованы с помощью генотипирования TaqMan.

Результаты: *Anopheles coluzzii* был основным разносчиком, обнаруженным в Нджамене (100%) и Массакори (~ 94%). В 147 насосавшихся крови F_0 *An. coluzzii* (82 из Нджамены и 65 из Массакори) не было найдено малярийного плазмодия. Высокоинтенсивная пиретроидная резистентность наблюдалась при условиях смертности < 2% для перметрина, дельтаметрина и этофенпрокса, и < 50% и < 60% после воздействия 10-кратных диагностических доз дельтаметрина и перметрина, соответственно. Для обоих участков смертность < 10% наблюдалась при ДДТ. Синергические биологические анализы с пиперонилбутоксидом значительно восстановили восприимчивость к

пиретроиду в популяциях Массакори с участием CYP450s (смертность = 13,6% для перметрина, $\chi^2 = 22.8$, df = 1, $P = 0.0006$; смертность = 13,0% для дельтаметрина, $\chi^2 = 8.8$, df = 1, $P < 0.00031$). Биоанализ с конусом ВОЗ по пиретроидам установил полную потерю эффективности противомоскитных сеток длительного применения (LLINs) и 100% восстановление восприимчивости после воздействия PermaNet® 3.0, содержащего пиперонилбутоксид. Обе популяции были восприимчивы к малатиону, но высокая устойчивость к бендиокарбу наблюдалась у популяции Массакори. Отсутствие мутации *ace-1* указывает на роль метаболической резистентности в устойчивости к бендиокарбу. Мутации 1014F и 1014S были обнаружены в обеих популяциях на уровне около 60% и < 20% соответственно. Секвенсирование интрона-1 натриевого канала, управляемого напряжением, выявило низкое генетическое разнообразие, свидетельствующее о снижении полиморфизма.

Выводы: высокая устойчивость к инсектицидам у чадских *An. coluzzii* подчеркивает проблемы, связанные с развертыванием противомоскитных сеток длительного применения (LLINs) и опрыскиванием внутри помещений (IRS) в сахеле. LLIN пиретроида-синергиста (например, PermaNet® 3.0) и IRS на основе фосфорорганических соединений могут стать альтернативами при борьбе с малярией в этом регионе.

Translated from English version into Russian by Maria Petrenko, revised by Alexander Somin, through

TRANSLATORS
WITHOUT BORDERS

Alta resistencia insecticida en el principal vector de la malaria *Anopheles coluzzii*, en la República de Chad

Sulaiman S. Ibrahim, Amen N. Fadel, Magellan Tchouakui, Ebai Terence, Murielle J. Wondji, Micareme Tchoupo, Clement Kérah-Hinzoumbé, Samuel Wanji, and Charles S. Wondj

Resumen

Contexto: la región Sahel, de la República de Chad, es el candidato principal para la pre-eliminación de la malaria. Para facilitar los esfuerzos de pre-eliminación en esta región, se carazterizaron dos poblaciones de *Anopheles coluzzii* desde la República del Chad Central , su perfil de resistencia a los insecticidas y los posibles mecanismos moleculares conduciendo una resistencia en el campo investigado.

Metodologías: se recolectaron hembras hematófagas de *Anopheles gambiae* s.l. las cuales descansaban en interiores se recogieron en N'djamena y Massakory, Chad, en 2018, y se caracterizaron para establecer la composición de la especie; también se determinó el índice de infección, a través de ensayos TaqMan. Se evaluó la susceptibilidad a varios insecticidas, utilizando bio-ensayos de tubos de la OMS. Se realizaron bioensayos de cono, utilizando varios mosquiteros tratados con insecticidas de larga duración (LLINs, por sus siglas en inglés). Se analizaron los resultados mediante pruebas de Chi al Cuadrado. Se investigó la resistencia desmontable (*kdr*) y los marcadores *ace-1*, por el genotipo TaqMan.

Resultados: *Anopheles coluzzii* fue el principal vector encontrado en N'djamena (100%) and Massakory (~94%). No *se encontró Plasmodium* en ninguna de las 147 hematófagas F_0 *An. coluzzii* (82 de N'djamena y 65 de Massakory). Se observó una alta intensidad de resistencia a piretroides, con una mortalidad de < 2% por permetrina, deltametrina y etofenprox, y con < 50% and < 60% de mortalidad, siguiendo a una exposición para dosis de diagnóstico 10x de deltametrina y permetrina, respectivamente. En ambos lugares, se observó < 10% de mortalidad por DDT. Los bioensayos sinérgicos con piperonilbutóxido recuperaron, significativamente, la susceptibilidad de los piretroides recuperados, , en las poblaciones de Massakory, lo cual implica CYP450s (mortalidad 13,6% para la permetrina, $\chi^2 = 22,8$, df = 1, $P = 0,0006$; mortalidad = 13,0% por deltametrina, $\chi^2 = 8,8$, df = 1, $P < 0,00031$). Los ensayos de cono-bioensayos establecieron la pérdida completa

de eficacia de los LLIN, basados en piretroides; y una recuperación del 100% de la susceptibilidad, tras la exposición al techo de PermaNet®3,0, que contiene piperonilbutóxido. Ambas poblaciones fueron susceptibles a malathion, pero se observó una alta resistencia de diviocarb, en la población Massakory. La ausencia de mutación *ace-1* señala el rol de la resistencia metabólica, en la resistencia al bendiocarb. se encontraron en ambas poblaciones, tanto la mutación 1014F como la 1014S , en aproximadamente del 60% y < 20%, respectivamente. La secuenciación del intrón-1, del canal de sodio dependiente de voltaje, reveló una baja diversidad genética, lo cual sugiere un polimorfismo reducido.

Conclusiones: la resistencia múltiple de las poblaciones de *An. coluzzii*, en Chad, resalta los desafíos relacionados con la implementación de los LLINs y la fumigación de interiores con efecto residual (IRS, por sus siglas en inglés), en el Sahel de este país. Los LLIN sinérgicos piretroides (por ejemplo, PermaNet®3.0) y el IRS, a base de organofosforados, pueden ser las alternativas para el control de la malaria, en esta región.

Translated from English version into Spanish by Victoria Marcé, revised by Maria Luz Puerta, through

TRANSLATORS
WITHOUT BORDERS
