# Supplementary figures and images for: High insecticide resistance in the major malaria vector Anopheles coluzzii in Chad Republic
Source: Infect Dis Poverty. 2019 Dec 3;8:100. doi: 10.1186/s40249-019-0605-x (PMC6892245; doi:10.1186/s40249-019-0605-x)

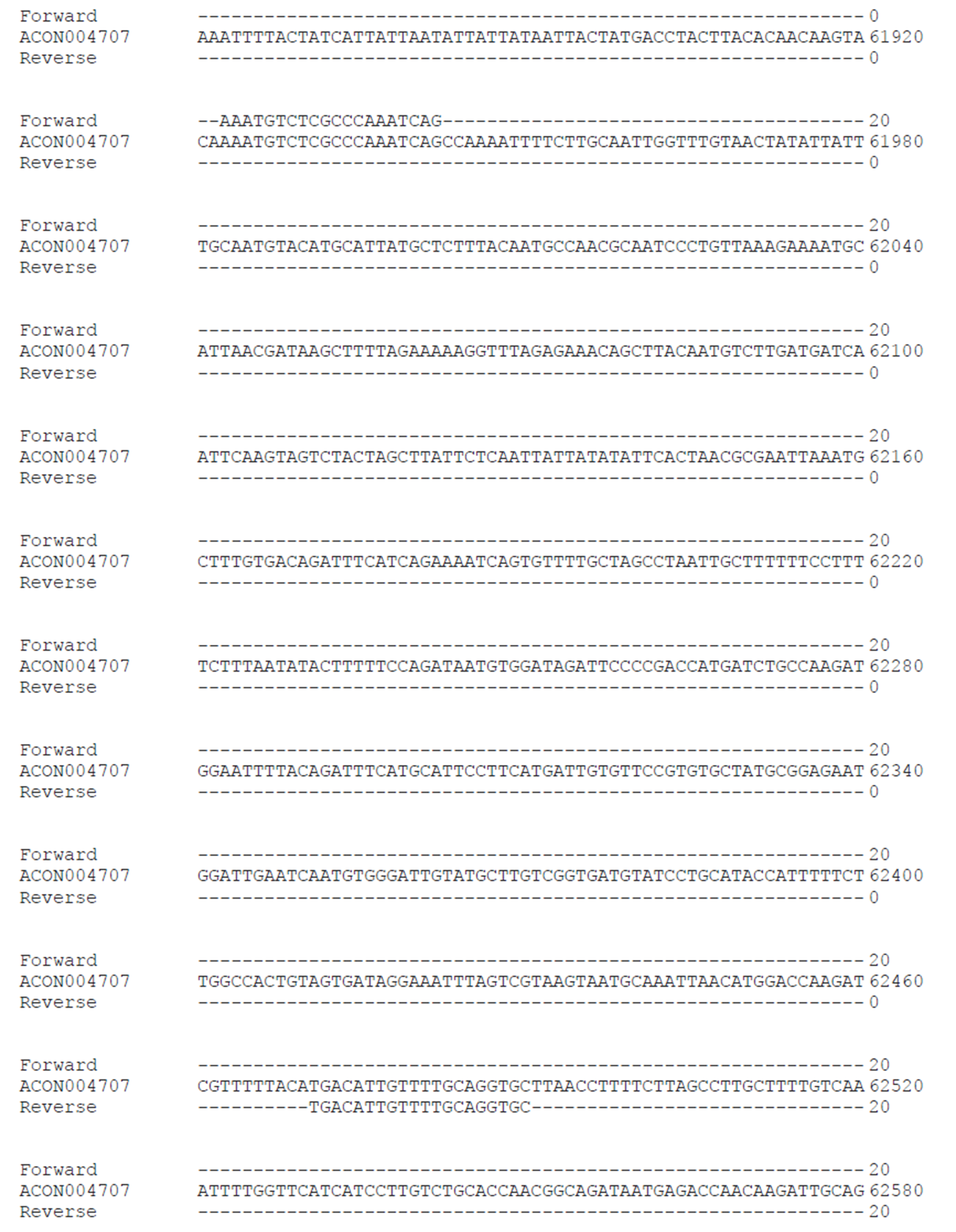

Supplement: Supplementary file 2 — Additional file 2: Figure S1. The nucleotide sequences of the voltage-gated sodium channel fragment, spanning the kdr locus. [file 40249_2019_605_MOESM2_ESM.tif]
